# 

**Published:** 1899-05

**Authors:** 


### PERSONALS.

Dr. William Herbert Lowe has been unanimously re-elected city veterinarian of Paterson, N. J. Dr. Lowe has a record of ten years' service as city veterinarian, and one that any practitioner might well feel proud of. Every member of the Board of Aldermen, without regard to party affiliations, voted for his reappointment.

Dr. F. C. Hutton, of Welland, Ont., was one of the judges of harness horses at the Toronto horse-show.

Dr. J. C. Bartholomew, of Berwyn, Pa., was a visitor to the Chesterbrook races early in May.

Dr. Otto von Laing, of Philadelphia, has been an inmate of the University Hospital for a number of weeks, combating a serious illness.

Dr. D. E. Salmon, of Washington, D. C., was a visitor to the Keystone Veterinary Medical Association meeting in Philadelphia in May.

Drs. Pearson, C. J. Marshall, and Hoskins, of Philadelphia, were in conference with Mayor Ashbridge, of the "City of Brotherly Love," in May, relative to an improvement in the meat-inspection and milk-inspection service.

Prof. Andrew Smith, of Toronto, Canada, was one of the judges of the pony classes under the saddle at the Boston horse-show.

Dr. James Marshall, of Philadelphia, takes an active interest in horses of speed, and has been selected as judge and starter at a number of tracks during the season just opening.

Dr. Alexander Glass, of Philadelphia, after an arduous season's work, will seek rest and recreation on his naphtha launch, which he recently overhauled and which now rests on the placid waters of the Delaware River.

Dr. R. L. Tritton, of Richmond, Va., is an admirer of the steeple-chase hunters, and exhibits his representative of this class in the shows.

Dr. C. A. Dohan, of Darling, Pa., was among those who were winners at the Chesterbrook races, riding his own horses in the steeple-chase classes.

Dr. F. C. Grenside, of New York, will be one of the judges at the horse-show of the Brooklyn Riding and Driving Club.

Joseph Shanor, V.S., of Beaver Falls, Pa., whose registration and practising in the State was declared illegal by the Pennsylvania State Board of Veterinary Medical Examiners, and the aid of the courts invoked to compel his ceasing to so do, has terminated by his removal to Salem, Ohio, and the maintenance of the law.

Dr. W. B. Switzer, of Williamson, N. Y., has located at Oswego in the same State.

Dr. Emil Knight, of Rochester, N. Y., was on the sick-list during the holidays.

Drs. Joseph Hughes, Chicago, Ill.; W. L. Williams, Ithaca, N. Y.; J. I. Gibson, Denison, Iowa, and C. P. Lovejoy of Princeton, Ill., were among those who addressed the convention of breeders at Chicago, in March, relative to the examination of stallions and breeding horses intended for exhibition at the Paris Exposition in 1900. The National Horse Breeders, Dealers, and Exhibitors' Association are planning for a grand exhibit at the coming exposition.

Veterinarian E. E. Sayers, of Algona, Iowa, after a four-years' record of faithful services in City Councils, has been called to the Mayoralty chair of his city, where the larger field of public usefulness as a good citizen will redound to the honor of the veterinary profession.

Dr. George B. Blackman, of Rome, Georgia, has removed to Blackman, Tennessee.

Dr. H. J. McClellan, formerly of Bryn Mawr, Pa., is now practising human medicine at Elliott, Illinois.

Dr. J. C. Foelker, of Allentown, Pa., was a visitor to Philadelphia in March, spending several days among his professional friends, visiting, among others, Drs. Thomas B. and James B. Rayner, E. M. Ranck, and others.

Dr. A. J. Sheldon, of Boston, Mass., was a visitor to New York, Philadelphia, and Lakewood, N J., during the Easter holidays.

Dr. W. H. Yingst, of Harrisburg, Pa., was on the sick-list the early part of April.

Dr. B. F. Senseman, of Philadelphia, spent the Easter holidays at his old home in Mechanicsburg, Pa.

Dr. W. W. Martin, of Philadelphia, has succeeded Dr. John H. McNeal as resident surgeon of the Veterinary Department of the University of Pennsylvania.

Dr. R. L. Tritton, of Richmond, Va., will act as one of the judges at the Philadelphia open-air horse-show on the class of horses, cobs, and ponies under saddle.

Dr. S. B. Willard, of Yardley, Pa., was appointed justice of the peace by the Governor of Pennsylvania.

Dr. W. G. Huyett, of Chicago, Ill., is temporarily sojourning at Wernersville, Pa.

Dr. T. D. Hinebauch, of North Dakota, has received the appointment of veterinarian for the third judicial district of that State. In addition to extensive interests in the breeding of thoroughbred stock, the doctor has some eight hundred and twenty-seven acres of ground under cultivation, and seems to be in thoroughly congenial work.

Dr. S. G. Burkholder, formerly of Denver, Pa., is now practising human medicine in Chicago, and teaching comparative medicine at Howard Medical College, and meat-inspection and milk-inspection at McKillip Veterinary College.

The many friends of Dr. Thomas B. Rayner, of Philadelphia, will be glad to learn of his rapidly improving health and return to his old-time activity.

Dr. C. P. Lyman, of Boston, has been on a sojourn to the Bermudas in search of renewed health for his wife and self. The loss of a married daughter during the past winter was a severe blow to the parents.

Dr. G. A. Wehr, of Denver, Pa., graduate of the Ontario Veterinary College, was among the list of graduates of the McKillip Veterinary College in March.

Dr. W. E. Wight, of Delaware, Ohio, member of the Ohio State Board of Veterinary Medical Examiners, was among the list of graduates of 1899 at the McKillip Veterinary College. Dr. Wight is also a graduate of the Ontario Veterinary College.

Dr. J. H. McNeal, resident surgeon of the Veterinary Department of the University of Pennsylvania, has received an appointment under the Bureau of Animal Industry and been assigned to duty at Buffalo, N. Y.

Dr. Louis A. Klein, formerly of Philadelphia, is now located at Fort Worth, Texas, in the quarantine-line service of the Bureau of Animal Industry.

Dr. John Graessl, of Covington, Ky., will visit Germany this summer and spend some months in his native country.

Little Opal Conkey, daughter of Dean L. L. Conkey, of the Grand Rapids Veterinary College, is but four years old, yet she can name and locate every bone in the equine body, as well as describe many of them. Dr. Conkey claims for her the distinction of being the youngest osteologist in America, perhaps in the world.

Dr. L. A. Merrillat, of Chicago, has been assiduous in his devotion to the securing of legislation on behalf of the profession of Illinois at the recent session of the State Legislature.

Dr. E. A. A. Grange, of Detroit, has been keenly interested in the efforts to obtain legislation regulating the practice of veterinary science in Michigan during the past winter.

Dr. D. E. Salmon, of Washington, and W. S. Devoe, of Chicago, were among those called to Washington to give testimony in the embalmed beef inquiry.

Dr. W. A. Heck was transferred from St. Louis, Mo., to Sioux City, Iowa, during the month of April.

Dr. John J. Maher, of Philadelphia, has opened a commodious veterinary hospital, with ample accommodation for some thirty equine patients, a large canine department, and a very attractive and unique dog ambulance.

Dr. S. D. Burkholder has resigned from the Bureau of Animal Industry to accept a permanent position on the teaching staff of the McKillip Veterinary College.

Dr. W. E. A. Wyman, a contributor to the Department of Surgery, enjoys a lucrative practice in Milwaukee.

Dr. H. B. Shugar, of Lebanon, Pa., formerly an assistant of Dr. E. B. Ackerman, of Brooklyn, was a visitor to the JOURNAL office early in May.

Dr. William H. Lowe, of Paterson, N. J., has completed the improvements and enlargement of his hospital, which add much to its convenience and adaptation to the work sought to be performed in connection therewith.

Dr. B. Royer has removed from Birnamwood to Shawano, Wisconsin.

Dr. W. W. Thorburn, of Lansing, Michigan, is proprietor of the Lansing creamery, which he conducts as an adjunct to his professional work.

Dr. R. A. Ramsey, of Mexico, Mo., has been appointed a meat-inspector and is now stationed at South Omaha, Neb.

Dr. Jos. M. Good, of Chattanooga, Tenn., has entered the service of the Bureau of Animal Industry, and is located at St. Joseph, Mo.

